# Arthroscopic Management of Spinoglenoid Cyst Compressing on the Suprascapular Nerve: A Case Report

**DOI:** 10.7759/cureus.110788

**Published:** 2026-06-13

**Authors:** O T George, Nithin Thomas George

**Affiliations:** 1 Orthopedics and Traumatology, St. Mary's Hospital, Thodupuzha, IND

**Keywords:** arthroscopic decompression, infraspinatus atrophy, muscle atrophy, spinoglenoid cyst, supra scapular nerve

## Abstract

Spinoglenoid cysts are a rare cause of suprascapular nerve compression and may present with shoulder pain, weakness, and muscle atrophy. Compression of the inferior branch of the suprascapular nerve at the spinoglenoid notch characteristically results in isolated infraspinatus weakness and atrophy, often leading to diagnostic delay due to nonspecific clinical symptoms. Magnetic resonance imaging is the modality of choice for identifying the location, size, and extent of the lesion. Arthroscopic decompression is considered the preferred treatment approach. We report the case of a 42-year-old male patient who presented with right shoulder pain, isolated weakness of external rotation, and infraspinatus muscle atrophy of three months duration. MRI revealed a spinoglenoid ganglion cyst causing suprascapular nerve compression. The patient underwent arthroscopic decompression of the cyst, resulting in immediate postoperative pain relief. A structured rehabilitation program was initiated, beginning with range-of-motion exercises followed by progressive strengthening. At follow-up, the patient demonstrated significant improvement in shoulder function. Spinoglenoid cysts are frequently overlooked or misdiagnosed because of vague shoulder complaints; however, they can lead to significant pain, weakness, and muscle atrophy if untreated. Arthroscopic decompression offers effective symptom relief and facilitates recovery of shoulder strength and range of motion.

## Introduction

Spinoglenoid cysts are an uncommon but important cause of shoulder pain and suprascapular nerve compression at the spinoglenoid notch. Compression at this location typically spares the supraspinatus muscle while selectively affecting the motor branch to the infraspinatus, resulting in isolated weakness or atrophy of the muscle [1]. Suprascapular nerve entrapment was first described by Kopell and Thompson in 1959 [2]. Patients may present with vague posterior shoulder discomfort, decreased external rotation strength, and functional impairment, making diagnosis challenging and frequently delayed. These cysts are frequently associated with labral pathology and may present with nonspecific posterior shoulder pain, often leading to delayed diagnosis [3]. Early recognition is essential, as prolonged nerve compression may result in irreversible denervation and persistent functional deficit [4]. Increased awareness of this condition and its characteristic clinical and imaging features is essential for timely diagnosis and appropriate management. We present a case of a spinoglenoid cyst causing suprascapular nerve compression with infraspinatus weakness.

## Case presentation

**Figure 1:**
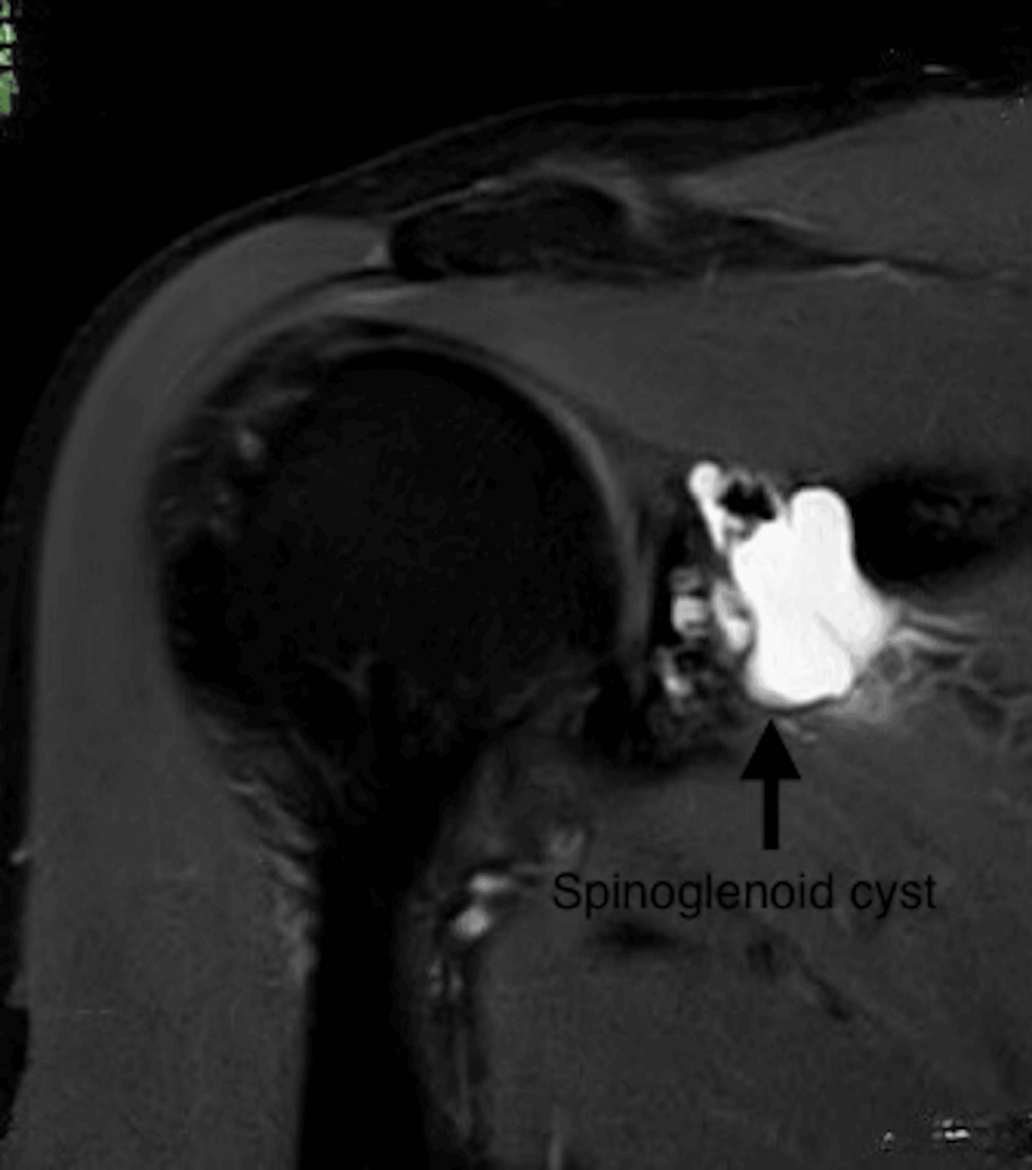
MRI of the right shoulder showing cystic lesion at spinoglenoid notch

**Figure 2:**
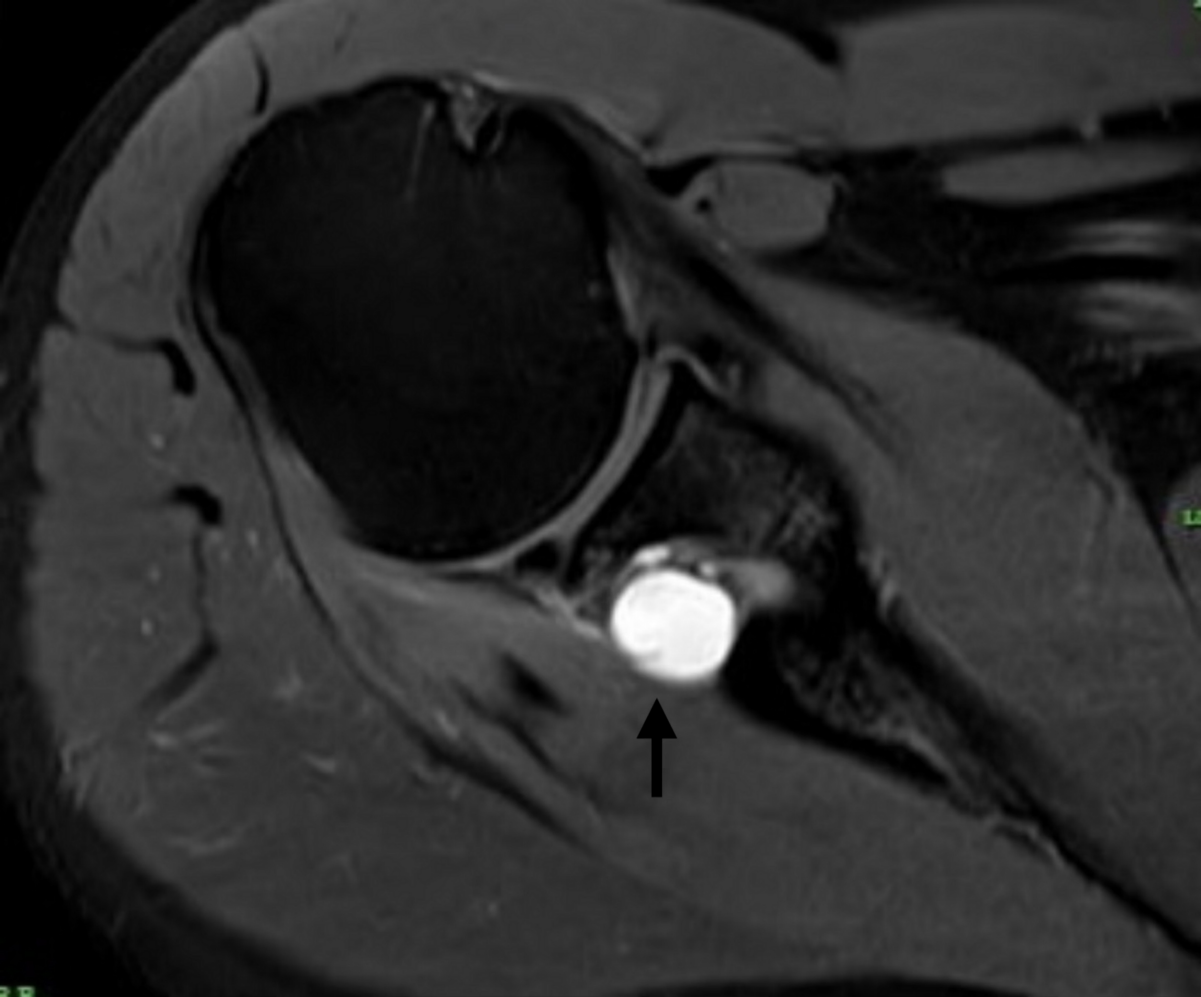
Axial cut of the right shoulder MRI showing spinoglenoid cyst (arrow)

**Figure 3:**
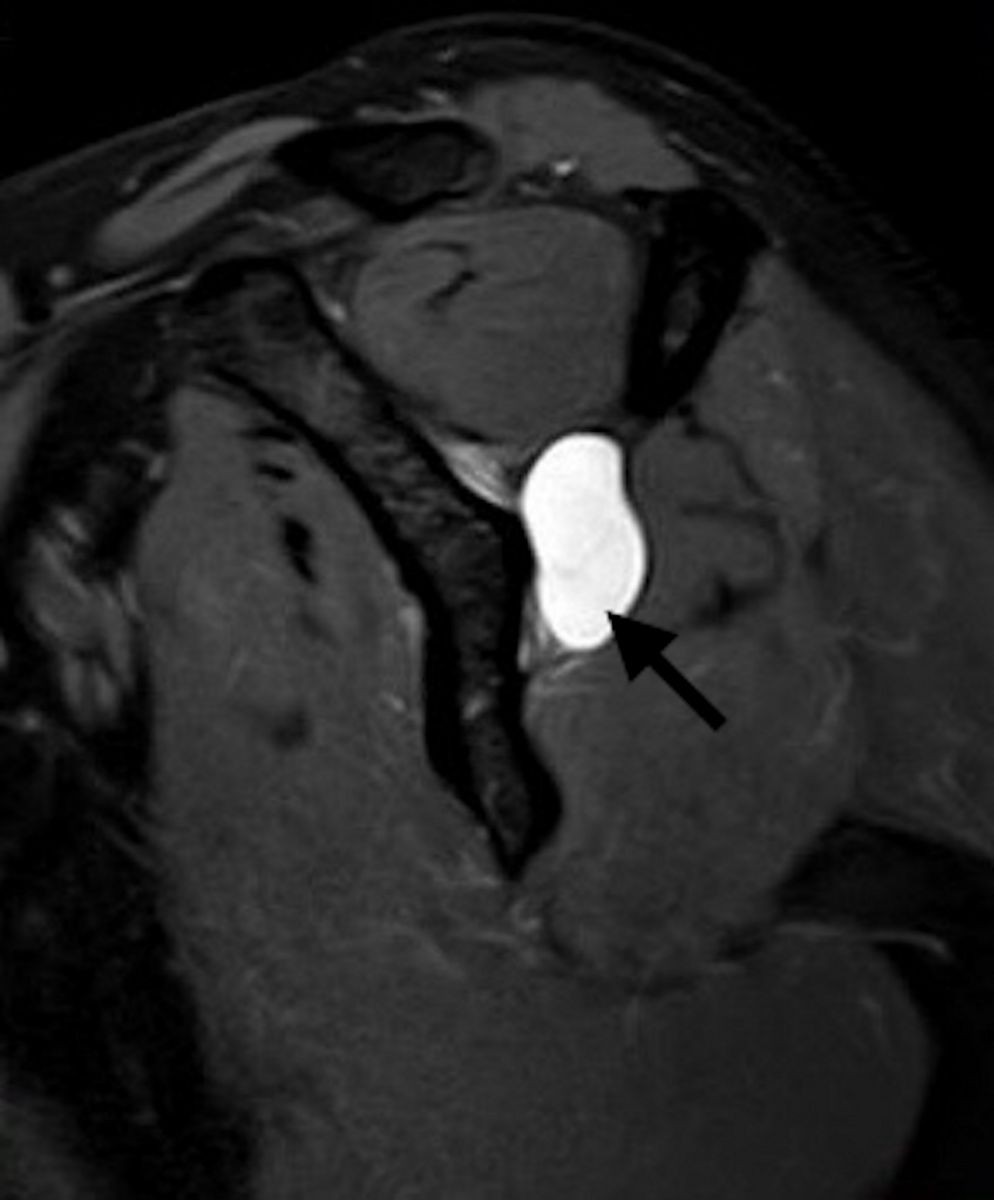
MRI of the right shoulder showing spinoglenoid cyst (arrow)

A 42-year-old male patient presented with a three-month history of right shoulder pain associated with isolated external rotation weakness and visible infraspinatus muscle atrophy. Magnetic resonance imaging revealed a spinoglenoid ganglion cyst (Figures 1-3).

The patient was placed in the beach chair position under general anesthesia. The affected upper limb was prepared and draped in a sterile manner, and a spider arm holder was mounted to hold the patient's limb in position. A standard posterior viewing portal was established. Diagnostic arthroscopy was done. An anterior working portal was created through the rotator interval under direct visualization. An accessory posterolateral portal was made.

**Figure 4:**
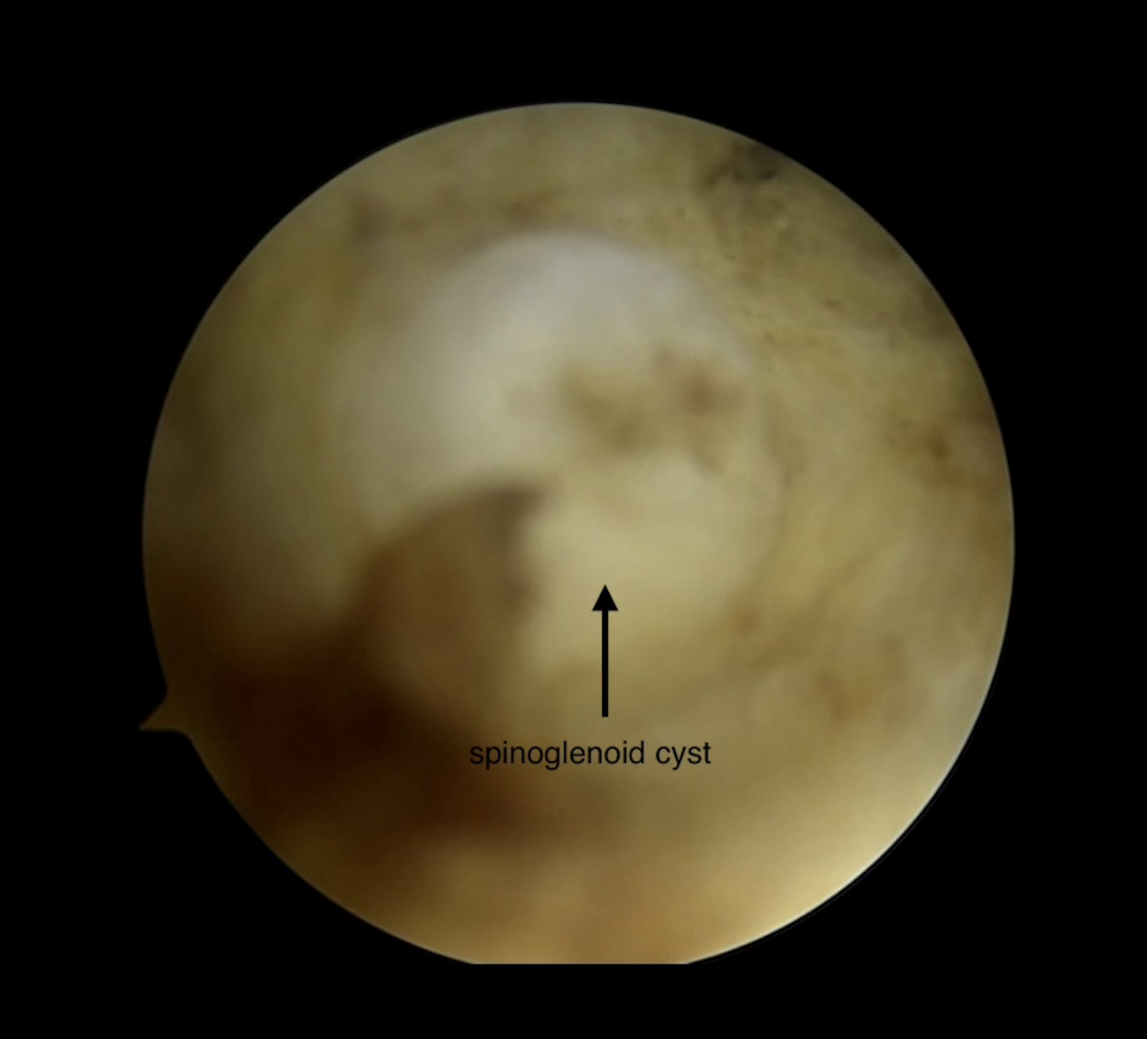
Arthroscopic visualization of spinoglenoid cyst

**Figure 5:**
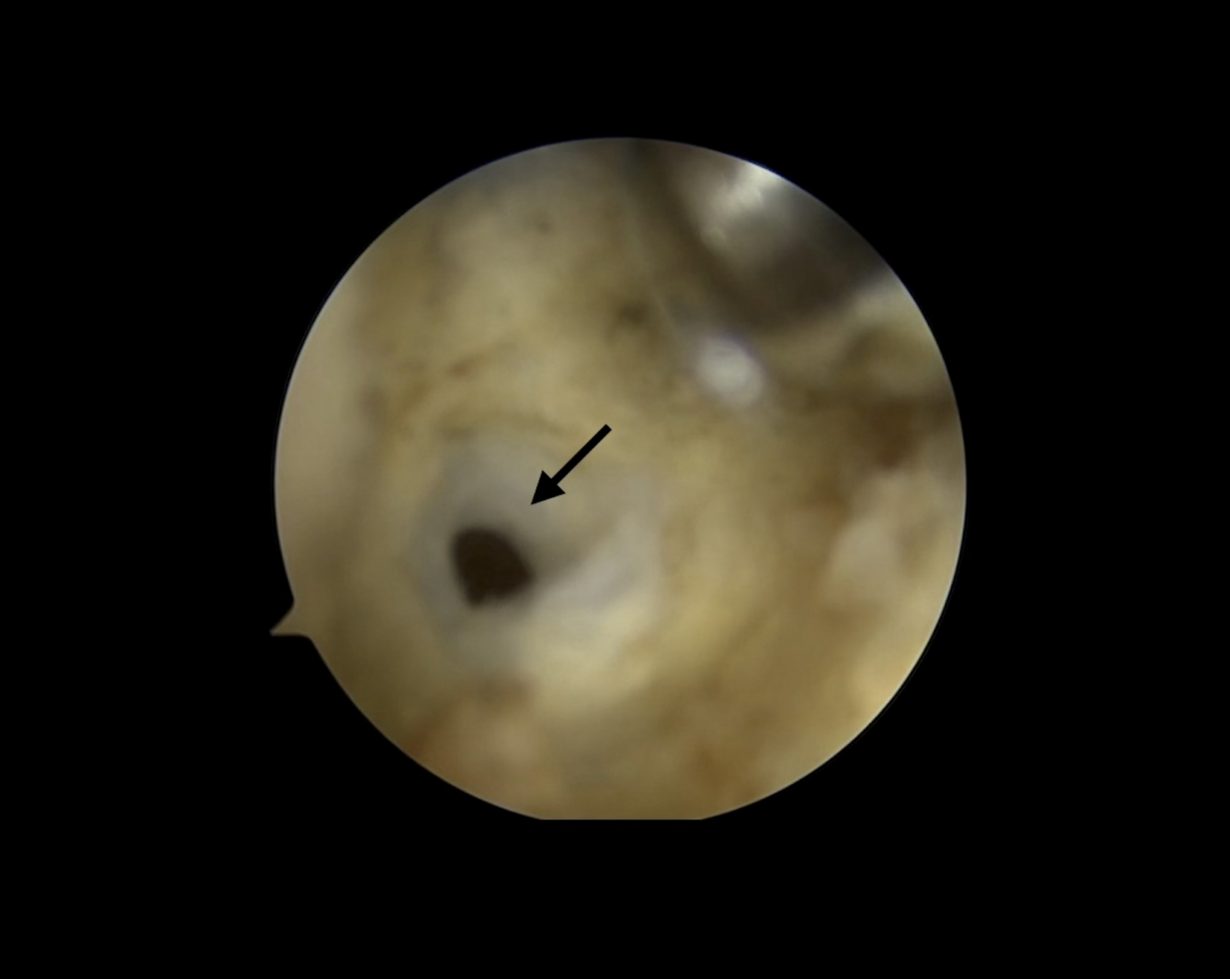
Arthroscopic visualization of cavity following the removal of spinoglenoid cyst (arrow)

**Figure 6:**
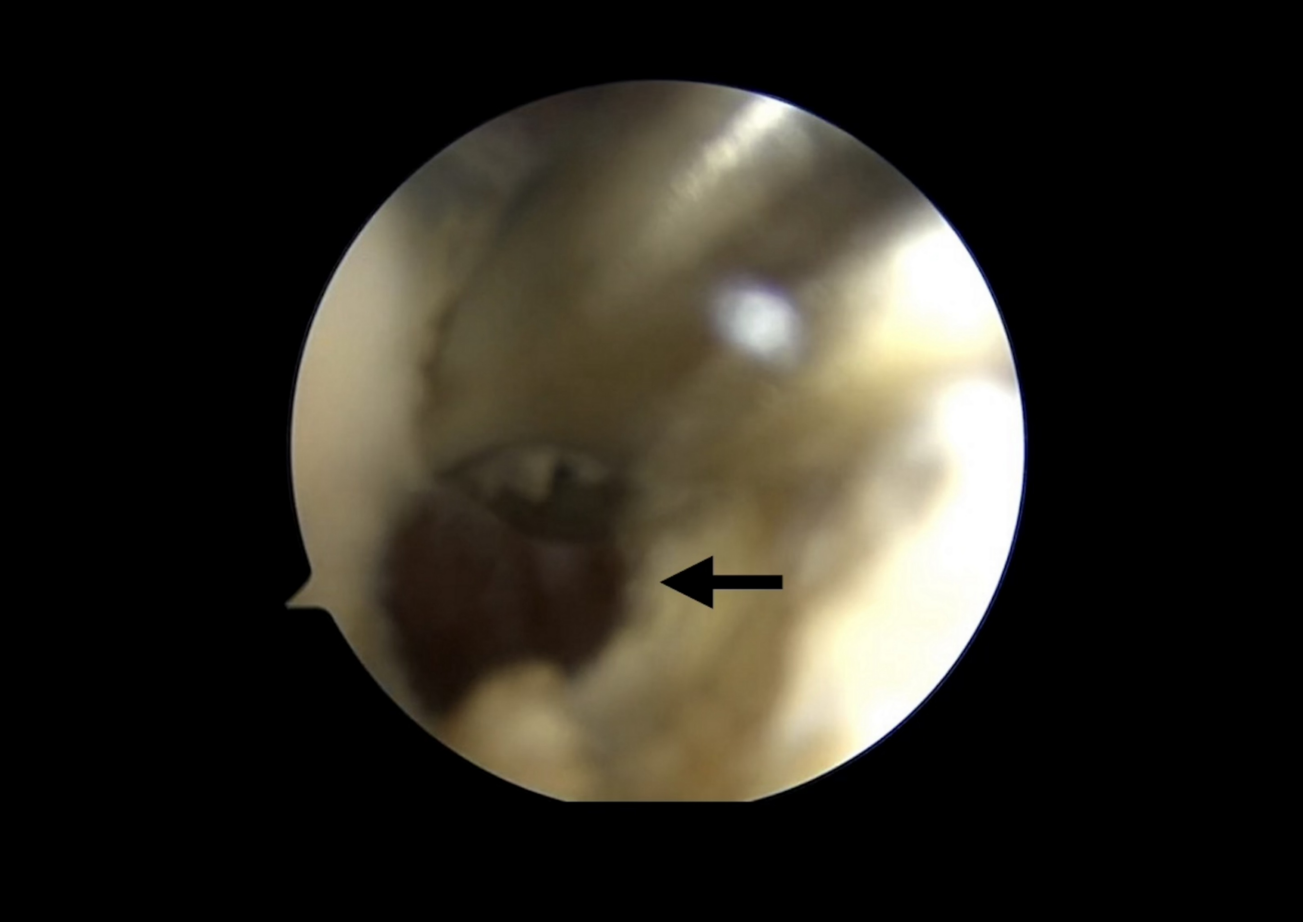
Arthroscopic visualization of the removal of remnants of cyst using shaver (arrow)

A translabral approach was utilized to access the spinoglenoid cyst. The posteroinferior labrum was carefully probed. A small, controlled labral split was made. A spinoglenoid cyst was identified (Figure 4). Using a shaver, the cyst was excised (Figure 5). Mucinous fluid was evacuated from the cyst. A shaver was used to completely remove the remnants of the cyst wall to prevent recurrence (Figure 6).

**Figure 7:**
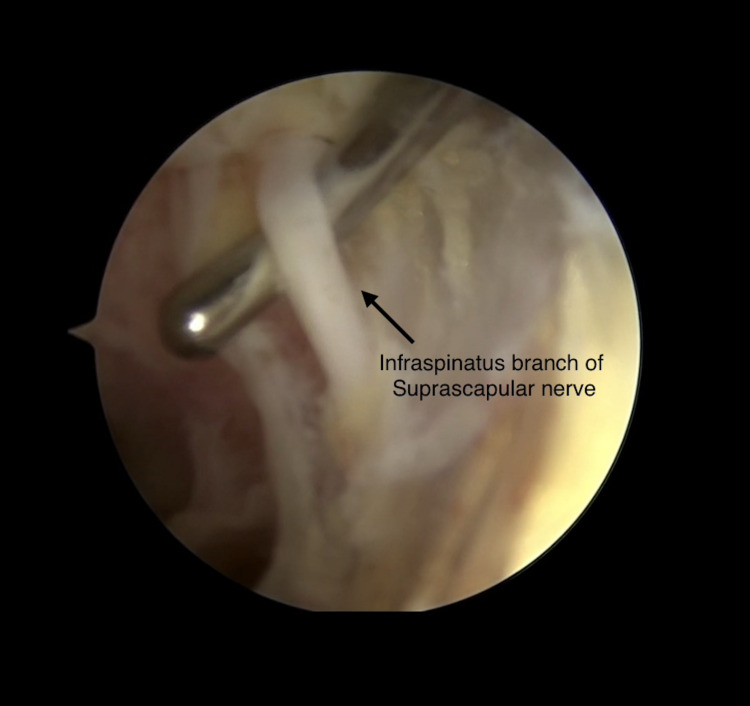
Arthroscopic visualization of the infraspinatus branch of the suprascapular nerve

Dissection was continued posteriorly to identify the infraspinatus branch of the suprascapular nerve (Figure 7). The nerve was visualized and gently isolated. No evidence of nerve transection or severe fibrosis was noted. Extreme caution was exercised to avoid traction or thermal injury. The labral split was left unrepaired due to minimal disruption and stable labral tissue.

Postoperatively, the arm was placed in a sling. Early passive and active-assisted range of motion exercises were initiated, followed by a progressive strengthening program. The patient reported significant pain relief immediately postsurgery. Gradual improvement in external rotation strength was noted. At the six-month follow-up, there was complete resolution of symptoms with no residual muscle wasting.

## Discussion

Spinoglenoid ganglion cysts are an uncommon cause of suprascapular nerve compression, leading to pain, isolated external rotation weakness, and infraspinatus muscle atrophy [5].The suprascapular nerve has motor innervation to the supraspinatus and infraspinatus muscles, but entrapment specifically at the spinoglenoid notch results in isolated infraspinatus dysfunction, helping to distinguish it from suprascapular notch lesions that affect both muscles [6].

The most widely accepted hypothesis for cyst formation is the "one-way valve" mechanism, where joint fluid escapes through a labral tear and accumulates along the path of least resistance into the spinoglenoid notch, compressing the nerve [7,8]. MRI is the diagnostic modality of choice, allowing clear visualization of the cyst and associated denervation changes in the infraspinatus muscle [6,9]. Electromyography (EMG) and nerve conduction studies can further confirm suprascapular nerve dysfunction and help quantify the degree of denervation [9].

Clinically, patients present with vague posterolateral shoulder pain worsened by abduction and external rotation. Weakness in external rotation accompanied by infraspinatus atrophy should prompt evaluation for a spinoglenoid cyst, as these signs are frequently misinterpreted as rotator cuff pathology or cervical spondylosis, which can delay diagnosis [6,9].

Treatment strategies for spinoglenoid ganglion cysts range from conservative measures (rest, physical therapy, activity modification, nonsteroidal anti-inflammatory drugs (NSAIDs)) to interventional approaches. Simple cyst aspiration under ultrasound or CT guidance has been reported; however, recurrence rates are high, and neurologic recovery may be incomplete when the underlying cause is not addressed [9].

Surgical decompression remains the definitive treatment for symptomatic cysts, especially when nerve compression and neurologic deficits are present. Arthroscopic decompression and cyst excision have become the preferred techniques due to less morbidity, direct visualization of intra-articular pathology, and the ability to simultaneously repair associated labral tears [5,6].

Arthroscopic series report high rates of pain relief and functional improvement following cyst decompression. In a series of 14 patients treated arthroscopically for spinoglenoid ganglion cysts with suprascapular neuropathy, all patients demonstrated improved external rotation strength and no clinical recurrence at a mean follow-up of 51 months [6]. Similar findings of pain relief and return of function have been described in multiple smaller case series [5,6]. Although literature supports arthroscopic decompression as effective with low recurrence, the evidence is mainly derived from case series and retrospective reports, and high-level comparative studies are limited [5,6,9].

## Conclusions

Spinoglenoid ganglion cysts should be considered in patients presenting with posterior shoulder pain, isolated external rotation weakness, and infraspinatus atrophy. Early diagnosis with MRI and timely surgical decompression are essential to prevent irreversible suprascapular nerve damage. Arthroscopic excision provides effective pain relief, restoration of shoulder function, and favorable neurological recovery with minimal morbidity. Appropriate postoperative rehabilitation further contributes to excellent functional outcomes, as demonstrated in this case.

## References

[1] Wee TC, Wu CH (2018). Ultrasound-guided aspiration of a paralabral cyst at the spinoglenoid notch with suprascapular nerve compressive neuropathy. J Med Ultrasound.

[2] Thompson WA, Kopell HP (1959). Peripheral entrapment neuropathies of the upper extremity. N Engl J Med.

[3] Meng B, Zhang Z, Li W, Wang Q, Cao J (2025). Arthroscopic management of spinoglenoid notch cysts: with and without labral lesions. BMC Musculoskelet Disord.

[4] Plancher KD, Evely TB, Brite JE, Briggs KK, Petterson SC (2021). Endoscopic/arthroscopic decompression of the suprascapular nerve at the spinoglenoid notch: indications and surgical technique. JSES Rev Rep Tech.

[5] Piatt BE, Hawkins RJ, Fritz RC, Ho CP, Wolf E, Schickendantz M (2002). Clinical evaluation and treatment of spinoglenoid notch ganglion cysts. J Shoulder Elbow Surg.

[6] Kostretzis L, Theodoroudis I, Boutsiadis A, Papadakis N, Papadopoulos P (2017). Suprascapular nerve pathology: a review of the literature. Open Orthop J.

[7] Westerheide KJ, Dopirak RM, Karzel RP, Snyder SJ (2006). Suprascapular nerve palsy secondary to spinoglenoid cysts: results of arthroscopic treatment. Arthroscopy.

[8] Westerheide KJ, Karzel RP (2003). Ganglion cysts of the shoulder: technique of arthroscopic decompression and fixation of associated type II superior labral anterior to posterior lesions. Orthop Clin North Am.

[9] Martin SD, Warren RF, Martin TL, Kennedy K, O'Brien SJ, Wickiewicz TL (1997). Suprascapular neuropathy. Results of non-operative treatment. J Bone Joint Surg Am.

